# Fine-Mapping and Selective Sweep Analysis of QTL for Cold Tolerance in *Drosophila melanogaster*

**DOI:** 10.1534/g3.114.012757

**Published:** 2014-06-26

**Authors:** Ricardo Wilches, Susanne Voigt, Pablo Duchen, Stefan Laurent, Wolfgang Stephan

**Affiliations:** Section of Evolutionary Biology, Department of Biology II, Ludwig-Maximilian University of Munich, 82152 Planegg-Martinsried, Germany

**Keywords:** cold tolerance, QTL, fine-mapping, selective sweep, *brinker*

## Abstract

There is a growing interest in investigating the relationship between genes with signatures of natural selection and genes identified in QTL mapping studies using combined population and quantitative genetics approaches. We dissected an X-linked interval of 6.2 Mb, which contains two QTL underlying variation in chill coma recovery time (CCRT) in *Drosophila melanogaster* from temperate (European) and tropical (African) regions. This resulted in two relatively small regions of 131 kb and 124 kb. The latter one co-localizes with a very strong selective sweep in the European population. We examined the genes within and near the sweep region individually using gene expression analysis and *P*-element insertion lines. Of the genes overlapping with the sweep, none appears to be related to CCRT. However, we have identified a new candidate gene of CCRT, *brinker*, which is located just outside the sweep region and is inducible by cold stress. We discuss these results in light of recent population genetics theories on quantitative traits.

Quantitative genetics assumes that selection on an adaptive trait involves simultaneous selection at multiple loci contributing to this trait. This causes small to moderate allele frequency shifts at these loci (Barton and Keightley 2002). Therefore, adaptation does not require new mutations in the short-term. Instead, selection may use alleles that are found in the standing genetic variation (Pritchard and Di Rienzo 2010). Genome-wide data suggest that this quantitative genetic view is relevant (Mackay *et al.* 2012). In particular, association studies confirm that quantitative traits are typically polygenic.

However, there is the view that the molecular population genetics scenario of selective sweeps is also important in describing selection on quantitative traits. These sweeps may be caused by new mutations or low-frequency alleles from the standing variation. Empirical evidence for the occurrence of sweeps at QTL has been reported in studies of artificial selection, including the domestication of chickens (Rubin *et al.* 2010), dogs (Axelsson *et al.* 2013), pigs (Rubin *et al.* 2012), and cattle (Qanbari *et al.* 2014). Furthermore, there is growing evidence of sweeps associated with positive directional selection on quantitative traits in natural populations. Linnen *et al.* (2013) studied coat color adaptation in deer mice controlled by a single large gene that shows multiple signatures of sweeps. Incomplete sweeps in the enhancer region of the gene *ebony* have contributed to the darker color of the abdominal segments of high-altitude *Drosophila melanogaster* from Uganda (Pool and Aquadro 2007; Rebeiz *et al.* 2009). Sweeps have also been observed at the EDA locus in three-spine sticklebacks associated with the reduction of lateral plate armor in fresh water environments (Cano *et al.* 2006). In the common sunflower, selective sweeps have revealed candidate genes for adaptation to drought and salt tolerance (Kane and Rieseberg 2007).

Sophisticated methods have been developed to detect selective sweeps in a genome (Thornton *et al.* 2007; Pavlidis *et al.* 2008; Stephan 2010). In this study, we utilized these methods in combination with quantitative genetics tools to dissect a QTL interval for cold tolerance in *D. melanogaster*. Cold tolerance has been shown to be driven by environmental selection (Hoffmann *et al.* 2002; Schmidt *et al.* 2005) and to have a highly polygenic basis (Morgan and Mackay 2006; Norry *et al.* 2008; Svetec *et al.* 2011; Mackay *et al.* 2012). In a previous analysis, using strains from African (tropical) and European (temperate) regions, we have identified X-linked QTL controlling chill coma recovery time (CCRT), a proxy for cold tolerance (Svetec *et al.* 2011). Here, we chose one interval of size 6.2 Mb, which contains two QTL shared between both sexes, and fine-mapped it using quantitative complementation tests. This resulted in a 131-kb region and a 124-kb region. In the European population, a strong selective sweep co-localized with the 124-kb region. We then analyzed this region in detail using population genetics and gene expression analyses. We found that the genes within the selective sweep region are probably not related to the trait, but a gene (*brinker*) just outside the sweep is induced by cold stress.

## Materials and Methods

### Fly lines

**Figure 1:**
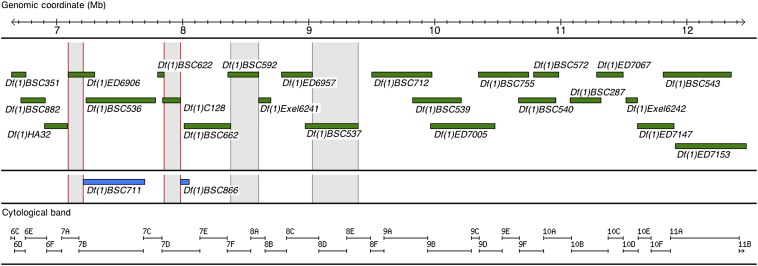
Map of tested deletions within the QTL interval undergoing study. All deletions, represented by green or blue bars, span a 5.8-Mb fraction of the 6.2-Mb-long interval of interest on the X-chromosome. Deletion breakpoints at the base pair level are known for all deletions except *Df(1)HA32* and *Df(1)C128*, for which only cytological bands are reported. Both physical and cytological coordinates are provided. The 24 deletions represented in green represent the minimum available set spanning the 5.8-Mb QTL interval; deletions in blue were tested on failure to complement of one of the overlapping deletions in green. Fractions of the QTL interval with light gray shading indicate regions of interest under deletions that show failure to complement. Red borders of this gray background indicate highly significant failure to complement (*P < *0.01).

To conduct quantitative complementation tests on chromosomal deletions, a set of available deficiency lines were ordered at the Bloomington stock center (http://flystocks.bio.indiana.edu). Although the QTL interval was defined by Svetec *et al.* (2011) to be 6.2 Mb long (6C to 11D), the set of available deletions spans 5.8 Mb of its total length between coordinates 6,642,419 and 12,461,494 that correspond to cytological bands 6C to 11B (Figure 1). This 5.8-Mb-long interval is covered by a total of 24 overlapping deletions with known breakpoints at the sequence level in 92% of the cases. Additional deficiencies were tested if necessary.

The African and European versions of the X-chromosome used in the complementation tests are contained in fly lines A* and E*, created by introgressing one X-chromosome from a population of Zimbabwe and one from the Netherlands into a common laboratory strain (Svetec *et al.* 2011). Hence, these two lines bear different X-linked alleles while the rest of the major nuclear chromosomes and mitochondrial DNA are the same. These two lines are the parents of an X-recombinant inbred population used to localize the QTL interval that concerns us in this project.

Prior to CCRT scoring experiments, virgin female flies bearing the deficiency chromosome and the respective balancer were mated with males of the A* and E* lines, respectively. Eggs were allowed to develop in the same medium in which they were laid at 23°. Female F1 were sorted on hatching by phenotype as balancer or deletion bearer. Because all balancer types used to maintain the deletions have a dominant mutation for eye shape at the *Bar* (*B^1^*) locus, F1 flies exhibiting the *B^1^* mutant phenotype were considered as balancer bearers, whereas wild-type appearance was indicative of bearing the deletion. Sorted flies were kept at room temperature until CCRT scoring on their fourth to sixth day of life.

Assessments of expression levels of candidate genes were conducted using 4- to 6-day-old female flies belonging to the Netherlands population (isofemale lines: NL01, NL12, NL14, NL15, NL16, NL18, NL19, NL20) and the Zimbabwean population (isofemale lines: ZK84, ZK131, ZK145, ZK157, ZK186, ZK229, ZK377, ZK398). Flies were reared at 23° and subjected to cold stress in the same manner as reported for CCRT scoring. Three flies per line were used as controls (not exposed to cold). Three flies of each line were snap-frozen in liquid nitrogen at 10 min after being brought to room temperature, whereas three remaining flies per line were scored for their CCRT and frozen 15 min after the minute in which they were reported as recovered. Control flies, which remained at 23° in glass vials during the 7 hr of treatment, were also snap-frozen at the end of this time period. Frozen material was stored at −80° until RNA extractions were performed. Population pools per line/treatment were made prior to RNA extraction. Each population pool per treatment consisted of eight flies of the same population. Three population pools per treatment were made for both the Netherlands and Zimbabwe.

### CCRT scoring

Once flies reached 4 to 6 days of age, they were scored for CCRT following the protocol of Svetec *et al.* (2011). Briefly, flies were transferred to glass vials without anesthesia and placed in an ice-water bath of 0° for 7 hr. At the end of this time period, flies were brought back to room temperature (23°) and observed in time intervals of 1 min. The minute in which a fly was standing on its feet was recorded as its CCRT.

### Quantitative complementation tests on deficiencies

On average, 35 female flies per each of the four resulting genotypes E*/def, A*/def, E*/bal, and A*/bal were scored. For ANOVA analysis, log-transformed CCRT scores per genotype, line (*L*), and genomic background (*G*) were kept as fixed effects. We focused on the significance of the interactions of these two factors (*L* × *G*) as well as on the following two conditions to call the procedure quantitative failure to complement: the differences in CCRT for the genotypes bearing the balancer had to be small compared with that of the genotypes bearing the deletion; and in the latter case, the E*/def flies should show reduced CCRT with respect to the A*/def genotypes. It is expected that if these conditions are satisfied, failures to complement due to the presence of other QTL outside the region in question can be ruled out. However, a failure to complement detected with a given deficiency either can be caused by its interaction with QTL alleles in the region uncovered by a deficiency (allelism) or arise by interaction between this deficiency and QTL alleles elsewhere in the genome (epistasis) (Pasyukova *et al.* 2000; Service 2004). Because we are interested in the allelic cases of failure to complement by using the E* and A* lines (as well as its inbred wild-type progenitor lines) in the tests, we control for most of the epistatic effects that can be caused by loci residing on chromosomes 2 and 3. Bonferroni correction was applied to control for multiple testing.

### RNA extraction and cDNA synthesis

RNA was extracted from pools using the MasterPure RNA Purification Kit (Epicentre Biotechnologies, Madison, WI), followed by DNase treatment. Purified RNA was quantified with a nanodrop apparatus and tested for genomic DNA contamination based on a PCR (Phusion) protocol using a primer pair binding in nontranscribed regions of the X-chromosome (Primer code: X-1435; sequence available on request). Samples tested positive for genomic DNA were excluded from downstream protocols. cDNA synthesis was performed with SuperScript III Reverse Transcriptase (Invitrogen, Carlsbad, CA) on 1400 ng of RNA per reaction.

### RT-qPCR assays

RT-qPCR assays for candidate genes *CG1958*, *CG1677*, *CG2059*, *unc-119*, *brk*, and *Atg5* were performed with primers designed using the online tool QuantPrime (www.quantprime.de) to match all possible transcript types per candidate gene. The ribosomal genes *RpS20* and *RpL32* were taken as reference genes, against which relative gene expression levels of our genes of interest were normalized. RT-qPCR assays consisted of a total of three biological replicates each run in triplicate and were conducted on a Real-Time thermal cycler CFX96 platform (BioRad, Hercules, CA). Each reaction was taken to a final volume of 10 µl using iQ SYBR Green Supermix (BioRad, Hercules, CA). Further details of the experimental setup, such as amplification efficiency assessments with dilution series, can be provided on request.

Obtained Cq values per replicate within line (or pool) and treatment were transformed to calibrated normalized relative quantities (CNRQ) following Hellemans *et al.* (2007). Log-transformed CNRQs were then used to test the hypothesis of expression differences between pairs of lines (or pool) within and between treatments. For this purpose, Welch two-sample *t*-tests were performed on comparisons with fold differences above a threshold (defined by the variance between technical replicates). The Benjamini and Hochberg (1995) *P*-value correction was applied to control for false positives, due to the high number of simultaneous tests performed.

### Basic population genetics analysis

Molecular variation was characterized in the genomic fragment uncovered by the deletion *Df(1)ED9606*; *i.e.*, a total of 124 kb between coordinates 7,089,000 and 7,212,999. Publically available whole-genome sequences generated by Illumina NGS technology for four *D. melanogaster* populations were retrieved from the Drosophila Population Genomics Project (DPGP) at http://www.dpgp.org. The populations include the Netherlands (NL) with 11 lines (NL01, NL02, NL11, NL12, NL13, NL14, NL15, NL16, NL17, NL18, NL19), France (FR) with 8 lines (FR14, FR151, FR180, FR207, FR217, FR229, FR310, FR361), Rwanda (RG) with 25 lines (RG2, RG3, RG4N, RG5, RG6N, RG7, RG8, RG9, RG10, RG11N, RG13N, RG15, RG18N, RG19, RG21N, RG22, RG24, RG25, RG28, RG2, RG32N, RG33, RG36, RG37N, RG38N), and Southeast Africa consisting of a pool of 18 lines from Zambia (ZI91, ZI261, ZI268, ZI468, ZO12, ZO65), Zimbabwe (ZK84, ZK131, ZK186, ZS5, ZS11, ZS56), and Malawi (MW6, MW11, MW28, MW38, MW46, MW63). The corresponding *D. simulans* sequence (Hu *et al.* 2013) was used as outgroup.

The following quality-control steps during the initial handling of the sequence data were used. First, nucleotides with a PHRED score lower than 21 were set to N. Unless otherwise stated, this quality criterion was applied to all analyses in which DPGP sequence data were used. Second, if a given polymorphic site in the alignment showed a frequency of N higher than 10%, then it was excluded from the analysis. The following summary statistics were then computed on 2-kb-long nonoverlapping windows: θ_π_ (Tajima 1983), θ_W_ (Watterson 1975), and divergence (*D_xy_*) to the outgroup (Nei 1987). In addition, pairwise *F*_ST_ was calculated as normalized distance of Nei (Nei and Li 1979). Neutrality tests based on the site frequency spectrum (SFS) using the Tajima (1989) *D* statistic were also calculated.

### Composite likelihood ratio test of positive selection

To investigate whether the observed SFS in the region of interest is compatible with the one expected after a selective sweep, we calculated the composite likelihood ratio (CLR) statistic (Kim and Stephan 2002; Nielsen *et al.* 2005; Pavlidis *et al.* 2010) as it is implemented in the software SweeD (Pavlidis *et al.* 2013). This likelihood ratio test statistic compares a selective sweep model and a neutral model that is calibrated with the genomic background frequency spectrum. We used the parallel version of the software (SweeD-P) to calculate the CLR statistic along the X-chromosome in our European sample (19 lines from the Netherlands and France). In addition to the classes of the SFS (*i.e.*, 1 to *n*-1, where *n* is the sample size), we considered two additional site classes consisting of sites that are monomorphic in the European sample and polymorphic in the Rwandan sample. Extending the SFS in this way was shown to improve the power of the method to detect selective sweeps (Nielsen *et al.* 2005). SweeD was run on a 16-core CPU using the command line option “- -monomorphic” with 500,000 grid points. The background SFS was taken from the complete X-chromosome. However, following Pool *et al.* (2012), we excluded from the analysis telomere and centromere regions of the X-chromosome due to their very low recombination rate. The coordinates of the excluded regions range from the origin until position 2,222,391 for the telomere and from position 20,054,556 to the end for the centromere region. Finally we compared the CLR profile of our region of interest to the profile calculated for the complete chromosome.

The significance level of the CLR-test statistic was calculated by simulating large genomic regions with the coalescent simulator fastsimcoal2 (Excoffier and Foll 2011) under a neutral model that takes into account our current knowledge of the demography of European populations of *D. melanogaster* (Laurent *et al.* 2011). For every one of the simulated datasets, we computed the CLR-test statistic in the same way as we did for the observed dataset and recorded the maximum CLR value. We used the 95^th^ quantile of the distribution of top CLR values as our significance threshold. Because this analysis becomes computationally intensive as the size of the simulated genomic region increases, we investigated the relation between the threshold value and the size of the simulated region. We simulated batches of 100 datasets of increasing size from 50 to 5000 kb in length and took the asymptotic value reached as the chromosomal threshold.

### *F*_ST_-based scan for positive selection

For the set of F_ST_ analyses performed with BayeScan (Foll and Gaggiotti 2008) (http://cmpg.unibe.ch/software/bayescan/), input files were prepared following the authors’ instructions. The different runs were performed using default parameters with the sequence data of the same DPGP samples from the Netherlands, France, Rwanda, and Southeast Africa. In addition, lines from the following African populations were included: Ethiopia (ED2, ED3, ED5N, ED6N, ED10N, EZ2, EZ5N, EZ9N, EZ25), Cameroon (CO1, CO2, CO4N, CO8N, CO9N, CO10N, CO13N, CO14, CO15N, CO16), and South Africa (SP80, SP173, SP188, SP221, SP235, SP241, SP254). SNP exclusion criteria were as follows: positions showing more than two segregating alleles as well as sites with less than 50% base calls in one population were excluded from the analysis.

## Results

We first describe the results of the deficiency mapping and then those of the population genetics analysis and the gene expression studies.

### Quantitative deficiency mapping

To dissect the QTL interval reported by Svetec *et al.* (2011) corresponding to the interval at 6C-11D of approximately 6.2 Mb in length, we conducted quantitative complementation tests for 24 overlapping deletions spanning 94% of this interval. The chromosome fractions comprising the remaining 6% of the interval were left untested due to lack of suitable deletions. With the set of 24 deficiencies (Figure 1), we could potentially uncover the effect of line-specific alleles (line-specific refers to the type of X-chromosome involved in the test, which is either African or European) at 588 (95%) of the 622 annotated and computationally predicted genes within the interval. Overall, 14 of 24 of the tested deficiencies showed significant line effects at the 5% level, whereas 9 of 24 showed a significant effect of the genomic background on CCRT scores (with the term “genomic background” we refer to the involved deletion and balancer chromosomes; see *Materials and Methods*). We observed failure to complement in 4 of the 24 tested deletions (Table 1). Failure to complement implied both a significant effect of line (*L*) and a significant line by genomic background interaction (*L* × *G*) as long as the differences in CCRT followed the expected direction. That is, there were shorter CCRT times for flies bearing the E* X-chromosome in the presence of the deletion compared with the corresponding flies bearing the A* X-chromosome in the presence of the same deletion, but no difference was shown between the CCRT of the flies bearing the E* and A* X-chromosomes in the presence of the balancer chromosome.

**Table 1:** Deficiency analysis of X-linked QTL affecting CCRT in female flies

		Mean CCRT (SD), min									
Deletion	Balancer	E*/Deletion	E*/Balancer	A*/Deletion	A*/Balancer	Δ*def*	*Δbal*	*P* L	*P* G	*P* L × G	Stock No.
*Df(1)BSC351*	*FM7h*	31.70 (7.99)	30.91 (7.88)	31.46 (9.42)	32.73 (8.72)	0.24	−1.82	0.070057	0.838897	0.343401	24375
*Df(1)BSC882*	*FM7h*	29.39 (9.94)	30.91 (7.88)	32.69 (12.41)	32.73 (8.72)	−3.3	−1.82	0.018773	0.149609	0.574953	30587
*Df(1)HA32*	*FM7c*	37.75 (8.24)	32.16 (9.01)	41.61 (10.42)	33.25 (9.28)	−3.86	−1.09	0.177930	0.000001	0.454433	947
*Df(1)ED6906*	*FM7h*	26.93 (5.66)	30.91 (7.88)	36.28 (8.52)	32.73 (8.72)	−9.35	−1.82	0.000103	0.779708	0.000289	8955
*Df(1)BSC711*	*FM7h*	35.73 (6.52)	30.91 (7.88)	34.03 (8.45)	32.73 (8.72)	1.7	−1.82	0.944120	0.011307	0.121501	26563
*Df(1)BSC536*	*FM7h*	36.27 (9.81)	30.91 (7.88)	36.43 (11.34)	32.73 (8.72)	−0.16	−1.82	0.055280	0.002486	0.473532	25064
*Df(1)BSC622*	*Binsinscy*	33.37 (9.22)	35.59 (8.83)	34.50 (8.65)	36.60 (9.59)	−1.13	−1.01	0.386305	0.121365	0.820123	25697
*Df(1)C128*	*FM6*	26.97 (6.16)	30.11 (8.63)	37.44 (8.11)	32.63 (7.74)	−10.48	−2.52	0.000012	0.606252	0.000885	949
*Df(1)BSC866*	*Binsinscy*	36.26 (8.16)	35.59 (8.83)	37.46 (11.06)	36.60 (9.59)	−1.21	−1.01	0.480645	0.501206	0.850165	29989
*Df(1)BSC662*	*Binsinscy*	40.29 (8.98)	35.59 (8.83)	39.89 (9.70)	36.60 (9.59)	0.39	−1.01	0.330040	0.002076	0.615274	26514
*Df(1)BSC592*	*Binsinscy*	31.42 (7.47)	35.59 (8.83)	37.92 (9.16)	36.60 (9.59)	−6.51	−1.01	0.063548	0.639100	0.031094	25426
*Df(1)Exel6241*	*FM7c*	31.57 (7.29)	32.16 (9.01)	30.29 (8.76)	33.25 (9.28)	1.29	−1.09	0.685768	0.224516	0.294193	7715
*Df(1)ED6957*	*FM7j*	27.90 (7.68)	32.16 (9.01)	29.21 (6.77)	33.25 (9.28)	−1.31	−1.09	0.261153	0.001693	0.795931	8033
*Df(1)BSC537*	*FM7h*	29.64 (7.36)	30.91 (7.88)	35.36 (8.17)	32.73 (8.72)	−5.71	−1.82	0.004959	0.528960	0.099098	25065
*Df(1)BSC712*	*FM7j*	40.21 (8.39)	35.59 (8.83)	39.66 (9.36)	36.60 (9.59)	0.55	−1.01	0.623921	0.005147	0.565518	26564
*Df(1)BSC539*	*Binsinscy*	31.50 (7.07)	35.59 (8.83)	34.30 (7.52)	36.60 (9.59)	−2.8	−1.01	0.239470	0.026941	0.414266	25067
*Df(1)ED7005*	*FM7h*	29.63 (6.45)	30.91 (7.88)	29.21 (6.46)	32.73 (8.72)	0.43	−1.82	0.060255	0.075504	0.350039	9153
*Df(1)BSC755*	*Binsinscy*	30.36 (7.49)	30.11 (8.63)	33.33 (6.92)	32.63 (7.74)	−2.98	−2.52	0.009057	0.547656	0.88738	26853
*Df(1)BSC540*	*FM7h*	32.20 (7.51)	30.91 (7.88)	34.73 (9.31)	32.73 (8.72)	−2.53	−1.82	0.022620	0.141156	0.845784	25068
*Df(1)BSC572*	*FM7h*	31.11 (6.04)	30.91 (7.88)	37.05 (7.54)	32.73 (8.72)	−5.94	−1.82	0.009129	0.070999	0.152617	25391
*Df(1)BSC287*	*Binsinscy*	36.11 (9.57)	35.59 (8.83)	35.23 (8.06)	36.60 (9.59)	0.87	−1.01	0.596120	0.852371	0.643564	23672
*Df(1)ED7067*	*FM7h*	28.89 (7.99)	30.91 (7.88)	29.58 (7.80)	32.73 (8.72)	−0.68	−1.82	0.039538	0.029350	0.702833	9154
*Df(1)Exel6242*	*FM7c*	33.96 (7.48)	32.16 (9.01)	32.93 (7.68)	33.25 (9.28)	1.03	−1.09	0.585559	0.397537	0.406384	7716
*Df(1)ED7147*	*FM7h*	31.43 (7.44)	30.91 (7.88)	34.96 (8.42)	32.73 (8.72)	−3.52	−1.82	0.010630	0.272976	0.497331	9171
*Df(1)BSC543*	*FM7h*	30.88 (6.43)	30.91 (7.88)	33.75 (6.67)	32.73 (8.72)	−2.88	−1.82	0.012423	0.689596	0.674376	25071
*Df(1)ED7153*	*FM7h*	29.80 (6.30)	30.91 (7.88)	30.47 (6.82)	32.73 (8.72)	−0.67	−1.82	0.028176	0.134114	0.603622	8953

Summary of quantitative deficiency tests performed with the listed deletions. Δ*def* is the difference between the average CCRT of flies bearing E* and A* chromosomes in the presence of a given deletion. Negative differences suggested the presence of CCRT reducing alleles at sites potentially uncovered by the deletion. Δ*bal* is the difference between the average CCRT of flies bearing E* and A* chromosomes in the presence of a given balancer chromosome. Note that deletions held with the same balancer show the same the Δ*bal* values. *P* L is the value for the line effect (E* or A*) from two-way ANOVA analysis. *P* G is the value for the genomic background effect (deletion or balancer) effect from two-way ANOVA analysis. *P* L × G P is the value for the interaction between the two aforementioned variables. Stock no. is the code number under which the fly line bearing the deletion can be ordered at the Bloomington Stock Center in Indiana.

Deletions *Df(1)ED6906* and *Df(1)C128* were the only ones that revealed a highly significant failure to complement. In the case of *Df(1)ED6906*, the difference between the means of the CCRT scores for the flies bearing this deletion is 9.18 min, whereas that of the flies harboring the balancer chromosome is 1.82 min (Table 1). This means that deletion *Df(1)ED6906* has potentially uncovered E* X-chromosome alleles and/or alleles with the opposite effect residing on the A* X-chromosome. A similar interpretation can be given for the results obtained for *Df(1)C128*, *Df(1)BSC592* and *Df(1)BSC537*, which also failed to complement, as evidenced by the significant *L* and *L* × *G* effects and by the higher CCRT differences in the presence of the deficiency than in the presence of the balancer. However, for the two last deletions, these effects were only marginally significant (Table 1). Thus, they are not considered for further study.

Although our results with *Df(1)ED6906* and *Df(1)C128* meet the requirements to be considered allelic failures to complement, these can also be interpreted as an epistatic failure to complement due to interactions of these deficiencies with other loci that affect CCRT residing elsewhere on the X or in the other two major chromosomes. We are aware of the limitation of quantitative deficiency mapping to tell these two causes apart (Pasyukova *et al.* 2000). This is also a problem in similar studies (Fanara *et al.* 2002; De Luca *et al.* 2003; Geiger-Thornsberry and Mackay 2004; Harbison *et al.* 2004). However, because we used the E* and A* lines that share the same genetic background for their respective wild-type–derived X-chromosomes and not the wild-type inbred lines NL14 and ZK157, we can exclude interactions with factors located outside the X-chromosome.

The fact that we used a set of overlapping deficiencies allowed us to better define the stretch that revealed quantitative failure to complement. With respect to the 210-kb-long deletion *Df(1)ED6906*, the 67.15 kb overlapping with deletion *Df(1)BSC536* were subtracted from the stretch of interest (Figure 1). Furthermore, the results of the complementation tests with yet another overlapping deficiency at the same end (*Df(1)BSC711*) allowed us to subtract an additional 19.64 kb from the 210 kb encompassing *Df(1)ED6906* (Figure 1). At the other end of deletion *Df(1)ED6906*, its overlap with deletion *Df(1)HA32* is not known at the base pair level. Thus, the redefined fraction of interest under deletion *Df(1)ED6906* encompasses 124 kb between coordinates 7.09 and 7.21 Mb. Similarly, for the other highly significant deletion *Df(1)C128*, the redefined region of interest has a of length 131 kb (between coordinates 7.85 and 7.98 Mb).

This quantitative complementation mapping approach based on overlapping deletions has allowed us to reduce the number of initial candidate genes within the QTL undergoing study from 622 to a subset of 21. A total of 7 genes are located within the 124 kb uncovered by deletion *Df(1)ED6906*, and 14 genes were uncovered by deletion *Df(1)C128*. This is remarkable given the substantial fraction of uncharacterized genes in the 6.2 Mb of the QTL defined by Svetec *et al.* (2011) and the absence of known *a priori* candidate genes for CCRT in this chromosomal region.

In the next section we show that of the two regions identified by the complementation tests, the 124-kb region uncovered by deletion *Df(1)ED6906* overlaps with a selective sweep identified in several previous analyses (Glinka *et al.* 2006; Boitard *et al.* 2012; Langley *et al.* 2012), whereas we did not detect evidence for positive directional selection in deletion *Df(1)C128* (see below). Therefore, in the following section we focus on deletion *Df(1)ED6906*.

### Molecular population genetics analysis

We characterized molecular variation in the genomic region of 124 kb uncovered by deletion *Df(1)ED9606* in natural populations of *D. melanogaster*. First, we calculated a set of summary statistics on a 2-kb nonoverlapping window basis using next-generation sequence data from two European (the Netherlands and France) and two African (Rwanda and Southeast Africa) populations. The Netherlands population and a set of Southeast African lines represent the gene pools from which the E* and A* lines were derived. The additional two populations consisted of French and Rwanda sequence data from the DPGP (Pool *et al.* 2012). These four populations allowed us to draw conclusions on patterns of variation in temperate and tropical populations.

For each population we obtained nucleotide diversity estimates measured by the average number of pairwise differences (θ_π_) and Watterson’s estimator (θ_W_). The European populations showed a three-fold to four-fold reduction in nucleotide diversity when compared with the African populations (Table 2). Supporting Information, Figure S1 depicts the values of the 2-kb windows along the entire region of 124 kb in the four populations. The θ_π_ values in the Netherlands and French populations are along a 40-kb fragment (positions 65,000 to 105,000) 1 SD lower than the average over the 124-kb region. This pattern is in contrast to that observed in the two African populations for the same coordinates, for which nucleotide diversity values tend to be above their respective population averages. *F*_ST_ estimates along the 124-kb region between each of the European populations and the Southeast African pool parallel the diversity estimates such that *F*_ST_ vales are higher where diversity is low in the two European populations (Figure S1). Regarding divergence of each population to *D. simulans*, the averages of the two European populations are approximately 10% whereas those of the African populations have values between 12% and 13% (Table 2).

**Table 2:** Summary statistics average for the QTL undergoing study in four *D. melanogaster* populations

Population	*θ*_π_, Mean (SD)	*θ*_W_, Mean (SD)	*D_xy_*, Mean (SD)	*Tajima’s D*, Mean (SD)
The Netherlands	0.0010 (0.0007)	0.0010 (0.0006)	0.0953 (0.0352)	−0.4995 (1.0403)
France	0.0008 (0.0007)	0.0008 (0.0006)	0.1042 (0.0382)	−0.1010 (0.869)
Rwanda	0.0031 (0.0011)	0.0041 (0.0013)	0.1286 (0.0422)	−0.9671 (0.4024)
Southeast Africa	0.0034 (0.0011)	0.0042 (0.0012)	0.1248 (0.0482)	−0.7834 (0.3875)

We also analyzed possible deviations from the standard neutral SFS using Tajima’s *D* statistic (Figure S2). The African samples from Rwanda and Southeast Africa show generally negative *D* values that are typical for these populations (Glinka *et al.* 2003; Pool *et al.* 2012). The European samples have average *D* values near zero and a larger variance, as was also found previously (Glinka *et al.* 2003). Most interestingly, however, is the 40-kb window from position 65,000 to 105,000, in which Tajima’s *D* has lower than average values (except for a peak around coordinate 87,000).

The patterns of polymorphism observed in the region of interest in the European and African populations revealed a conspicuous reduction of genetic variability and a negative Tajima’s *D* in both European populations that extends for approximately 40 kb between relative position 65,000 and 105,000 in the 124-kb region. This reduction already has been identified as a footprint of positive selection in non-African populations (Glinka *et al.* 2006; Boitard *et al.* 2012; Langley *et al.* 2012). In this work, motivated by the link to the QTL, we revisited these analyses.

**Figure 2:**
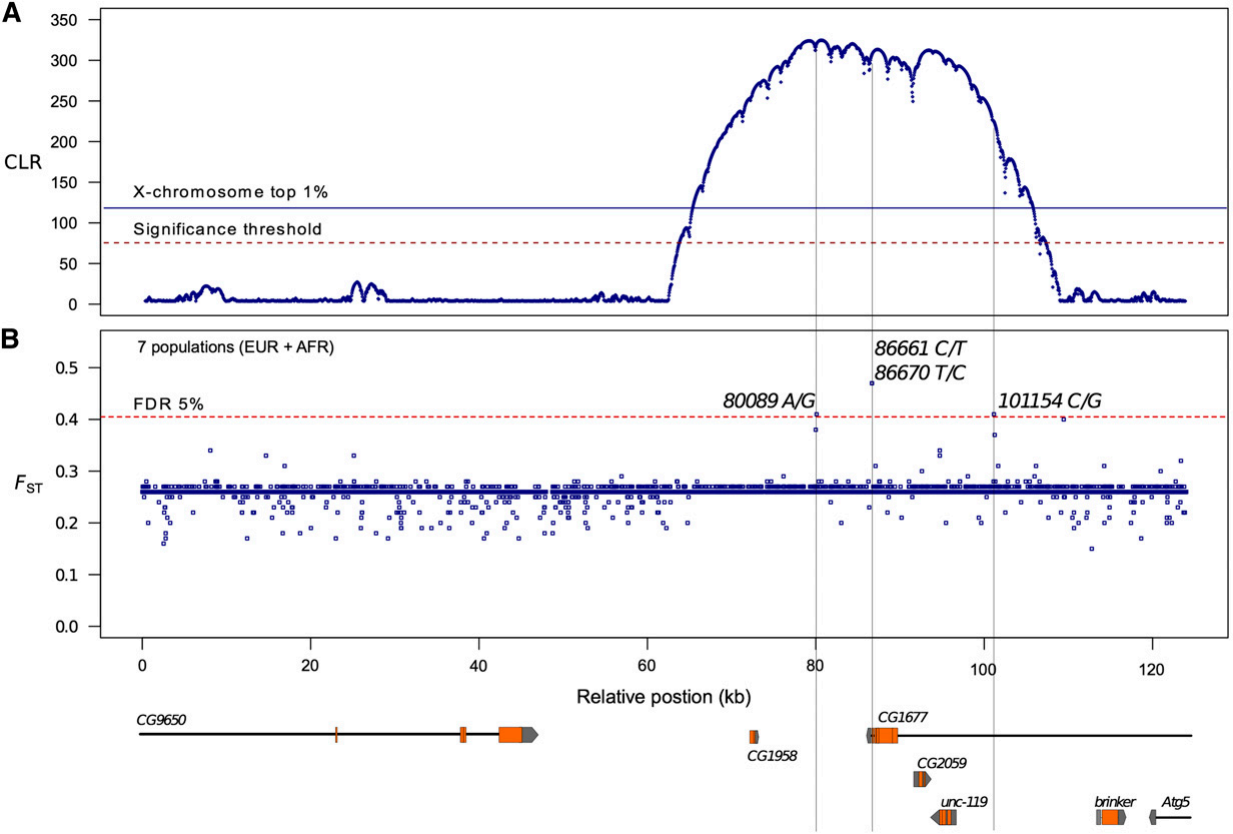
Evidence of positive selection and candidate SNPs in the 124-kb region under deletion *Df(1)ED6906*. (A) Likelihood (CLR) profile along the 124-kb on the X-chromosome using SNP data of two pooled European *D. melanogaster* from the Netherlands and France. Two significance thresholds are depicted. The solid line corresponds to the average of the top 1% CLR values for the X-chromosome in Europe and the dashed red line represents the significance threshold from simulations of equivalent subgenomic regions. (B) Model-based *F*_ST_ values for 7316 SNPs from a dataset including two European and five African samples (see *Materials and Methods*). Top SNPs (above the false discovery rate of 5%) are marked with position and a thin continuous line across panels.

Because demographic scenarios (particularly bottlenecks) can create similar genomic patterns as positive directional selection (Stephan and Li 2007), we subjected the data of the available European samples (pooling the Netherlands and French lines) to the most advanced composite likelihood ratio test (Pavlidis *et al.* 2013). This likelihood ratio was computed between a selective sweep model and a neutral model that is calibrated with the genomic background frequency spectrum. The background SFS was obtained from 20 Mb of the X-chromosome, excluding the telomere and centromere regions (see *Materials and Methods*). In our region of interest, the fragment between relative positions 65,000 and 105,000 exhibits a SFS that is in contrast to that of the genomic background and is better described by a selective sweep model (Figure 2A). The CLR values obtained for this interval (Λ_CLR_ >300) are within the top 1% of CLR values along the entire region of the X-chromosome analyzed (Figure S3) and are above the significance threshold of 72 that corresponds to the 95^th^ quantile of the top CLR values of 100 simulated subgenomic regions of 5 Mb. This value did not increase when larger genomic regions were simulated (Figure S4). Simulations were based on our current understanding of the demographic history of European populations (Laurent *et al.* 2011; Duchen *et al.* 2013).

Using the same CRL test, we also analyzed in detail *Df(1)C128*, the second highly significant deletion uncovered by the quantitative complementation test (between coordinates 7.85 and 7.98 Mb). However, we could not find evidence for selective sweeps (see Figure S3).

Because a large fraction of the region of low variation in Europe (particularly the coding regions of genes *CG1958*, *CG1677*, *CG5059*, and *unc-119*; see the gene model below) (Figure 2B) contains no or very little variation, the CLR tests cannot be used to identify the targets of selection. Instead, we utilized genetic differentiation between African and European populations to obtain model-based *F*_ST_ coefficients (Foll and Gaggiotti 2008; Riebler *et al.* 2008) for each SNP within the 124-kb region of interest (Figure 2B). We considered SNP data from seven populations along a gradient across Africa and Europe: South Africa, Southeast Africa, Rwanda, Cameroon, Ethiopia, France, and the Netherlands. Using BayeScan (Foll and Gaggiotti 2008), we obtained a pattern of *F*_ST_ values from a dataset of 7316 SNPs with an average *F*_ST_ of 0.2621 and revealed four outlier SNPs that show the highest differentiation across populations at an FDR of 5% (Figure 2B). These four SNPs are located within the 40-kb-long fragment enriched for SNPs showing significant CLR values between positions 65,000 and 105,000 (Figure 2A). The 65-kb-long and 19-kb-long flanking regions to the left and to the right of the 40-kb fragment, respectively, are enriched for SNPs showing below-average *F*_ST_ values (Figure 2B). However, none of these SNPs with low differentiation across populations is significant at the 5% FDR.

The exclusion of European populations from the analysis did not change the pattern of high-differentiation outlier SNPs (results not shown). This suggests that allele frequency differentiation at outlier SNPs had already started within the African continent. Furthermore, because the European populations probably have experienced more severe bottlenecks than the African populations (Li and Stephan 2006; Pool *et al.* 2012), we may conclude that the BayeScan results are relatively robust against demographic changes and are not due to the strong bottleneck in the European population.

**Figure 3:**
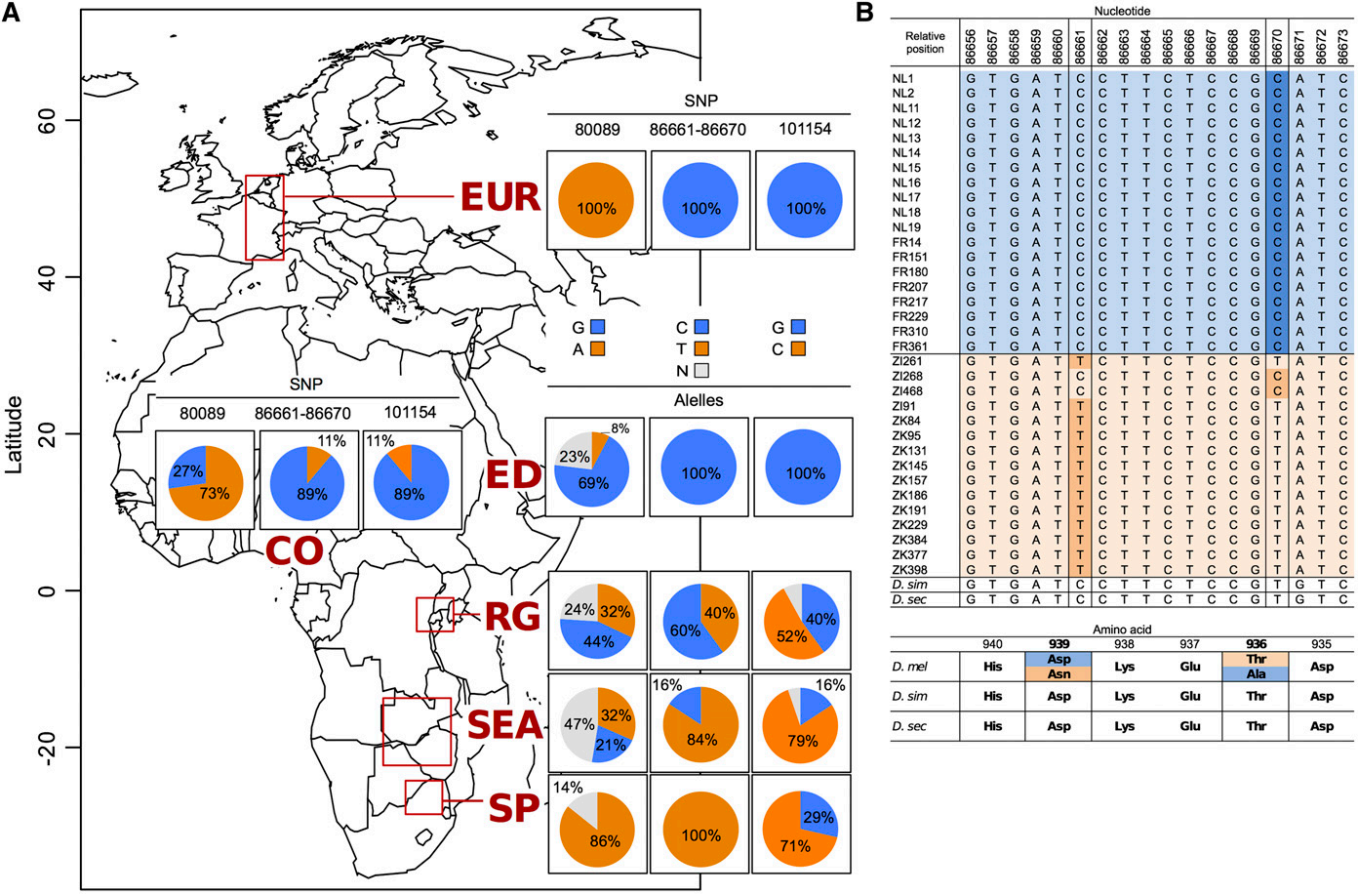
Allele frequency change at highly differentiated SNPs at the QTL of interest. (A) Allele frequencies of the top four highly differentiated SNPs across seven different populations along a latitudinal gradient. Populations are as follows: the Netherlands and France (EUR), Ethiopia (ED), Cameroon (CO), Rwanda (RG), Southeast Africa (SEA), and South Africa (SP) (see *Materials and Methods*). (B) European and Southeast African *D. melanogaster* haplotypes for the two nonsynonymous SNPs (86,661–86,670) in intron 5 of gene *CG1677*. These two SNPs correspond to amino acid positions 939 and 936.

Among the outlier SNPs that show high differentiation across the entire intercontinental dataset, the top ones are *86,661C /T* (*F*_ST_=0.4697, α=2.02, q-value=0.0024) and *86,670T/C* (*F*_ST_=0.4654, α=1.98, q-value=0.0042). These two nonsynonymous SNPs are located in exon 5 of the computationally predicted gene *CG1677* and show alleles in perfect linkage disequilibrium (LD) (Figure 3). The TT haplotype (*86,661T–86,670T)* is in high frequency in the Southeast African samples and is intermediate in Rwanda; its frequency decreases with increasing latitude to be replaced in the European populations by the CC haplotype. Both SNPs predict changes in the amino acid sequence of the protein. The common Southeast African form of the protein codes for a threonine (Thr) and an asparagine (Asn) at residues 936 and 939, respectively, whereas the cosmopolitan form has an alanine (Ala) and aspartic acid (Asp) at these two positions. The third highly significant SNP is *80,089A/G* (*F*_ST_=0.4146, α=1.54, q-value=0.0313) located between genes *CG1958* and *CG1677* (Figure 2). Its allele frequency distribution across populations is also shown in Figure 3. Finally, SNP *101,154C/G* (*F*_ST_ = 0.4068, α=1.4804, q-value = 0.0481) is located 5 kb upstream of gene *unc-119* within the large intron of gene *CG1677* (Figure 2).

### Candidate gene expression analyses and complementation tests with *P*-element insertion lines

**Figure 4:**
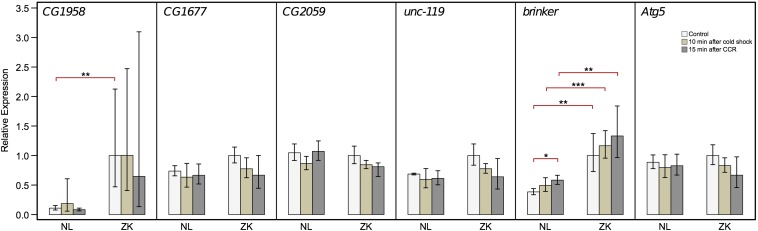
Expression of genes located in the region under deletion *Df(1)ED6906* that was affected by positive selection (see Figure 2A). Relative expression was measured under two cold stress and control conditions in pools of flies from a temperate population [the Netherlands (NL)] and a tropical population [Zimbabwe (ZK)]. Expression levels of these candidate genes were normalized with respect to that of the ribosomal genes *RpS20* and *RpL32*. The height of the bars represents the mean of three calibrated normalized relative quantities (CNRQ) per pool per gene rescaled to that of the corresponding ZK control. Error bars also represent rescaled confidence intervals. Levels of significance for tests of differences in expression levels among treatments within and between populations are indicated with asterisks: *P < 0.05; **P < 0.01; and ***P < 0.001.

We observed that the CLR profile of the selective sweep does not overlap with *brinker* and *Atg5*, but spans four of the seven candidate genes in the 124-kb region (see gene model below) (Figure 2B). To analyze whether these four genes in the sweep region are related to cold tolerance, we conducted expression analyses; *brinker* and *Atg5* were also included (Figure 4). qPCR assays were performed on cDNA prepared from pools of female flies from the Netherlands and Zimbabwe (see *Materials and Methods*). Expression of candidate genes was measured at two time points after cold stress exposure as well as under control conditions. The two post-cold stress time points were 10 min after the end of cold stress and 15 min after flies recovered from chill coma. Controls consisted of flies of the same lines that were not subjected to cold stress.

Of the six genes, *CG1958* and *brinker* showed significant differences in constitutive expression levels between the Netherlands pool and the Zimbabwean pool (*P <* 0.01) (Figure 4). This difference in expression levels between these populations also has been previously observed (Hutter *et al.* 2008). Furthermore, average expression level appeared to be unaffected by cold stress within pools at 10 min during recovery from chill coma for five of the genes. At this time point, the only highly significant difference between pools was observed for *brinker* (*P <* 0.001). Expression levels measured at 15 min after recovery from chill coma revealed one significant difference within pools: *brinker* was significantly overexpressed with respect to controls in the Netherlands pool (*P <* 0.05). Between-pool contrasts at 15 min after recovery from chill coma revealed only a significant difference for *brinker* (*P <* 0.01). This suggests that—of the six candidate genes—only *brinker* is induced by cold stress and may contribute to CCRT variation between temperate and tropical populations.

We also investigated *brinker* induction on the A* and E* lines individually. Again, we found that *brinker* is significantly induced 15 min after recovery from chill coma only in the E* line (*P* < 0.01). Furthermore, we noted that the constitutive expression differences between A* and E* disappeared (in contrast to the experiment with the African and the Netherlands pools described above), presumably because the A* and E* lines share the same autosomal background (data not shown).

Finally, we performed quantitative complementation tests on two of the four genes under the sweeps (*CG1677* and *unc-119*) for which lines with *P*-element insertions were available. None of these tests (performed in the same way as with the deletions) revealed quantitative failure to complement (Table 3). This further supports our conclusion that it is unlikely that the genes under the sweep (at least *CG1677* and *unc-119*) affect cold tolerance.

**Table 3:** P-element analysis of X-linked candidate genes affecting CCRT in female flies

		Mean CCRT (SD), min									
P-element	Balancer	E*/Mutation	E*/Balancer	A*/Mutation	A*/Balancer	Δ*mut*	*Δbal*	*P* L	*P* G	*P* L × G	Stock No.
*P(EPgy2)[52]CG1677EY06475*	*FM7a*	29.28 (8.92)	31.32 (9.48)	29.96 (8.73)	32.44 (10.16)	−0.67	−1.12	0.38070	0.02130	0.83404	17545
*P(EPgy2)unc-119EY20221*	FM7a*	27.99 (7.83)	31.32 (9.48)	31.78 (9.28)	32.44 (10.16)	−3.79	−1.12	0.01166	0.02542	0.15217	22375

Summary of quantitative deficiency tests performed with the listed P-element insertions. Δ*mut* is the difference between the average CCRT of flies bearing E* and A* chromosomes in the presence of a given P-element. Negative differences suggest the presence of CCRT reducing alleles at the gene affected by the tested P-element. The other symbols are defined in Table 1.

## Discussion

### Overview

First, we dissected a QTL interval for CCRT (a proxy for cold tolerance) using quantitative complementation tests. This approach revealed two deletions that failed to complement. Second, we used population genetic methods to narrow the number of genes in these two deletions. This approach led to the precise demarcation of a strong selective sweep in deletion *Df(1)ED6906*. Third, we investigated the genes within and near the sweep region by gene expression analysis. We found no evidence that the four genes within the sweep region (*CG1958*, *CG1677*, *CG2059*, and *unc-119*) are related to cold tolerance. However, this analysis also revealed a new candidate gene related to CCRT: *brinker*, a gene located just outside the sweep region that was induced by cold stress. In the following, we discuss these results, including the methods used.

### Quantitative complementation tests on deficiencies and gene expression assays

Using a set of deficiencies in the framework of the quantitative complementation test allowed us to narrow the QTL interval to two highly significant deficiencies, one of which contains a selective sweep. Furthermore, the list of genes under the QTL (encompassing the sweep) could be reduced to seven candidate genes. However, there is a caveat, because for both deletions significant *L* × *G* interactions were found in the presence of significant *L* effects. This is not uncommon in *D. melanogaster* (Fanara *et al.* 2002; De Luca *et al.* 2003; Geiger-Thornsberry and Mackay 2004; Harbison *et al.* 2004), but this means that it is difficult to distinguish between allelic failure to complement at the deficiency and an epistatic interaction between the deficiency and variation elsewhere. However, for the context of this article, this is not important because we have not relied exclusively on quantitative complementation tests to show relatedness to CCRT. In the case of *brinker*, our evidence of an association with CCRT is confirmed by gene expression analysis.

### The genes under the selective sweep and the putative targets of positive selection

Although we have not estimated the selection coefficient, the large value of the CLR statistic indicates that the selective sweep at deletion *Df(1)ED6906* in the European population is very strong, the strongest on the entire X-chromosome analyzed (see Figure 2 and Figure S3). It encompasses approximately 40 kb (with boundaries that are sharply defined). Based on the demarcation of the sweep, we observe that *brinker*, the only candidate gene that was induced by cold stress, is located outside the sweep. The four genes under the sweep are not induced in both African and European populations. *CG1958* is differentially expressed at the constitutive level (Figure 4), and it has been reported that *CG1677* expression is increased relative to constitutive levels during sustained cold stress (Graveley *et al.* 2011). Therefore, the expression of these genes may be temperature-dependent. However, it seems unlikely that regulatory elements determining their expression are the target of selection related to cold tolerance. Furthermore, we have performed quantitative complementation tests on two of the four genes under the sweep (*CG1677* and *unc-119*) for which lines with *P*-element insertions were available. None of these tests (performed in the same way as with the deletions) revealed quantitative failure to complement (Table 3). This supports our conclusion that it is unlikely that the genes under the sweep affect cold tolerance.

This leaves us to search for fixed differences in coding sequences on which selection for cold tolerance may have operated. To identify strong selective fixations (leading to sweeps) in coding regions, we need to analyze the sweep profile in more detail. Yet, because variation is almost completely depleted in this genomic region, we cannot use the CLR approach even if we include LD (Pavlidis *et al.* 2010). Instead, we used an *F*_ST_-based method (Foll and Gaggiotti 2008) to identify the target(s) of positive selection. The results are shown in Figure 2B.

We found four significantly differentiated polymorphisms under the selective sweep. The two SNPs that code for amino acid differences in the gene *CG1677* are most interesting. In the Southeast African sample, both combinations, Thr-Asn and Ala-Asp, are present at positions 936 and 939, where the former is more common. No other combinations exist. In Europe, however, Ala-Asp is fixed (Figure 3B). Subjecting the primary protein sequence encoded by this gene to a structure prediction program (Kelley and Sternberg 2009) reveals that both amino acid positions are part of the α-helix, *i.e.*, they are located on neighboring helix turns and can therefore interact. Interestingly, Thr and Asn can form one hydrogen bond between their side-chains more than Ala-Asp. The combination Thr-Asn may therefore make the protein more heat-stable than Ala-Asp (Perl and Schmid 2002), which appears to be an advantage in tropical Africa, given that ambient temperature is an important variant affecting life history traits in fruit flies. Conversely, the combination Ala-Asp may lead to a less rigid structure and thus possibly a more efficient protein, which may be an advantage in the temperate climate of Europe. Ancestral state reconstruction (Lewis 2001) shows that the Thr-Asn combination represents the ancestral state with high probability and that Ala-Asp arose through two point mutations. Because the intermediate states are not observed in the European and African population samples, the transition from Thr-Asn to Ala-Asp probably follows a compensatory evolution model (Kimura 1985; Innan and Stephan 2001) in which the intermediates are assumed to be strongly deleterious.

Do these adaptive fixations have anything to do with cold adaptation? The protein encoded by *CG1677* is part of the spliceosome (Herold *et al.* 2009) whose function may depend on temperature. However, there is no evidence known to us that splicing has a specific function in the protection of flies against cold. The other two significantly differentiated SNPs occur in noncoding regions between genes *CG1958* and *CG1677* and within the huge intron of *CG1677* (see gene model below) (Figure 2B). There is no evidence that they are involved in the regulation of cold tolerance. This leads us to conclude that strong positive selection causing the observed sweep has probably operated on traits (or molecular variants) other than cold tolerance.

### Comparison with population genetics theory

One of our salient observations is that the genes within the selective sweep region do not affect CCRT, whereas *brinker* located just outside the sweep is related to this trait. A similar observation was made previously for another QTL of cold tolerance in *D. melanogaster* (Svetec *et al.* 2011). Recent theoretical work has addressed the question of whether we should expect to find selective sweeps at genes controlling a quantitative trait. Chevin and Hospital (2008) presented a model for the footprint of selection at an adaptive QTL in the presence of background variation due to other loci. This analysis is based on the Lande (1983) model that consists of a locus of major effect on the trait and treats the remaining loci of minor effects as genetic background (such that background variation is maintained at a constant amount). This model predicts that adaptive traits that are under stabilizing selection and show the molecular signature of a selective sweep are only a very small subclass of quantitative traits. Pavlidis *et al.* (2012) analyzed a model with *n* loci controlling a trait under stabilizing selection. In their model, sweeps are more common than in the scenario presented by Chevin and Hospital (2008). They find that a multi-locus response to selection may in some cases prevent selective sweeps from being completed, but that conditions causing this to happen strongly depend on the genetic architecture of the trait. For instance, the probability of fixation of selected mutations decreases with the number *n* of loci involved and also depends on their effect sizes. Fixations are more common when the effects are approximately equal (in absolute size). This raises the question of to what extent CCRT is under stabilizing selection and to what extent CCRT is under directional selection. Although there is evidence that cold tolerance may have experienced positive directional selection from one optimum in Africa to another optimum in Europe, it is currently unclear whether this optimum shift is sufficiently large to overcome stabilizing selection that is expected to be widespread (*e.g.*, in the form of apparent stabilizing selection due to pleiotropic deleterious effects of mutations).

### *brinker*—a new candidate gene of CCRT

Based on our gene expression study (Figure 4), *brinker* is a candidate gene affecting variation in cold tolerance. However, it is important to note that *brinker* is located outside the large selective sweep described above and thus is not affected by the strong selection generating this sweep. This is consistent with current theory that sweeps at genes controlling phenotypic traits under stabilizing selection are expected to be rare (see above).

Theoretical models of weak selection (particularly for highly polygenic traits) predict the occurrence of allele frequency shifts between populations as a hallmark of polygenic selection (Hancock *et al.* 2010). For this reason, we searched the region upstream of *brinker* and found one conspicuous indel polymorphism (Figure S5A) at relative positions 109,442 to 109,976, *i.e.*, approximately 3 kb upstream of *brinker* and thus also outside the sweep region. This indel is located close to a polymorphic marker (Figure S5A) that is significantly associated with CCRT in a Raleigh population (Mackay *et al.* 2012). Using an extended sample of populations from the DGPG project (Pool *et al.* 2012), we investigated the frequencies of this indel polymorphism in these populations. We classified the indel polymorphism into nondeletion haplotypes and three classes of deletions (see Figure S5B). Based on linear regression analysis of the frequencies of the nondeletion haplotypes, we detected two antiparallel latitudinal clines where one spans from the populations near the equator (Rwanda, Gabon, Cameroon, Ethiopia, and Nigeria) to the north (France and the Netherlands) and another one from the equator to the south (Southeast Africa and South Africa) (*P* < 0.05 in both cases). This agrees with models of weak selection on highly polygenic traits. However, to what extent the observed frequency differences from the equator to the north and to the south explain the expression differences of *brinker* between tropical and temperate populations (see Figure 4) is currently an open question and beyond the scope of this article.

## Supplementary Material

Supporting Information
