# Supplementary material for: Fine-Mapping and Selective Sweep Analysis of QTL for Cold Tolerance in Drosophila melanogaster
Source: G3 (Bethesda). 2014 Jun 26;4(9):1635–45. doi: 10.1534/g3.114.012757 (PMC4169155; doi:10.1534/g3.114.012757)
Supplement: Supporting Information [file supp_4_9_1635__index.html]

Fine-Mapping and Selective Sweep Analysis of QTL for Cold Tolerance in Drosophila melanogaster — Supporting Information 

# Fine-Mapping and Selective Sweep Analysis of QTL for Cold Tolerance in *Drosophila melanogaster*

## Supporting Information for Wilches *et al.*, 2014

**Files in this Data Supplement:**

- Supporting Information - Figures S1-S6 (PDF, 647 KB)
- Figure S1 - Polymorphism and between-population differentiation along the 124 kb of interest. (PDF, 604 KB)
- Figure S2 - Tajima's *D* statistics. (PDF, 484 KB)
- Figure S3 - X-chromosome CLR profile for Europe. (PDF, 423 KB)
- Figure S4 - CLR thresholds vs. simulated fragment size. (PDF, 445 KB)
- Figure S5 - Putative *cis*-regulatory element upstream of *brinker*. (PDF, 555 KB)
