# Supplementary material for: Fine-Mapping and Selective Sweep Analysis of QTL for Cold Tolerance in Drosophila melanogaster
Source: G3 (Bethesda). 2014 Jun 26;4(9):1635–45. doi: 10.1534/g3.114.012757 (PMC4169155; doi:10.1534/g3.114.012757)
Supplement: Supporting Information [file supp_g3.114.012757_012757SI.pdf]

G3·Genes | Genomes | Genetics

Fine-mapping and selective sweep analysis of QTL for cold tolerance in *Drosophila melanogaster*

Ricardo Wilches*[1], Susanne Voigt*, Pablo Duchen*, Stefan Laurent* and Wolfgang Stephan*

*Section of Evolutionary Biology. Department of Biology II, Ludwig-Maximilian University of Munich. 82152 Planegg-Martinsried, Germany.

[1]Corresponding author: wilches@bio.lmu.de

DOI: 10.1534/g3.114.012757

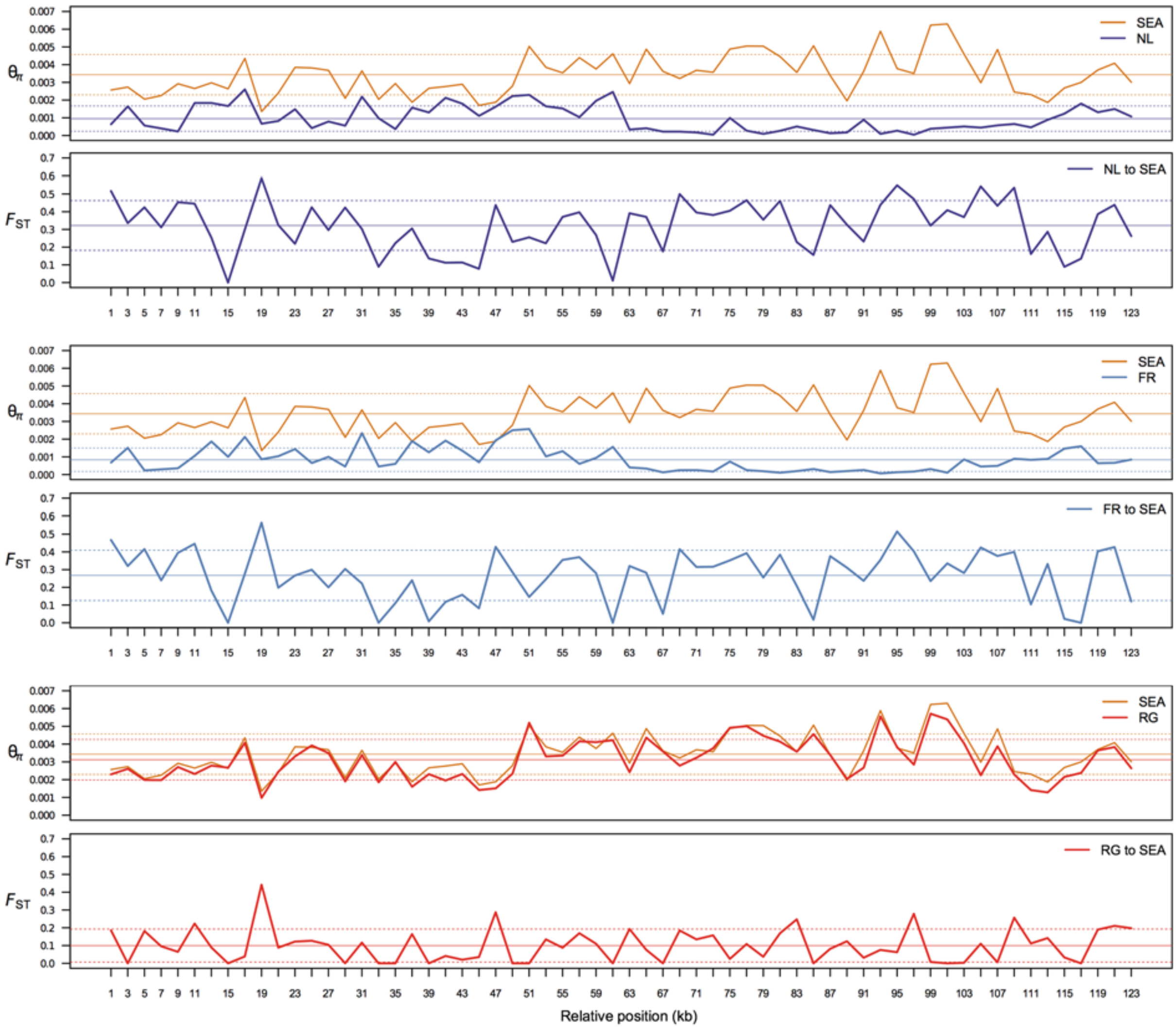

Figure S1 Polymorphism and between-population differentiation along the 124 kb of interest. Nucleotide diversity (θ_π) obtained for consecutive 2-kb long windows in four different populations: the Netherlands (NL), France (FR), Rwanda (RG) and a pool of Southeast African (SEA) lines sampled around Lake Kariba in Zimbawe and Zambia. This pool also includes lines from Malawi. The SEA profile is shown in all three θ_π panels for sake of comparison. Below each θ_π panel, inter-population differentiation profiles are plotted. Differentiation (F_{ST}) was calculated as normalized distance of Nei. Thin continous lines represent the average value for each summary statistic across the 62 windows, dashed lines represent 1 SD above and below the corresponding summary statistic mean.

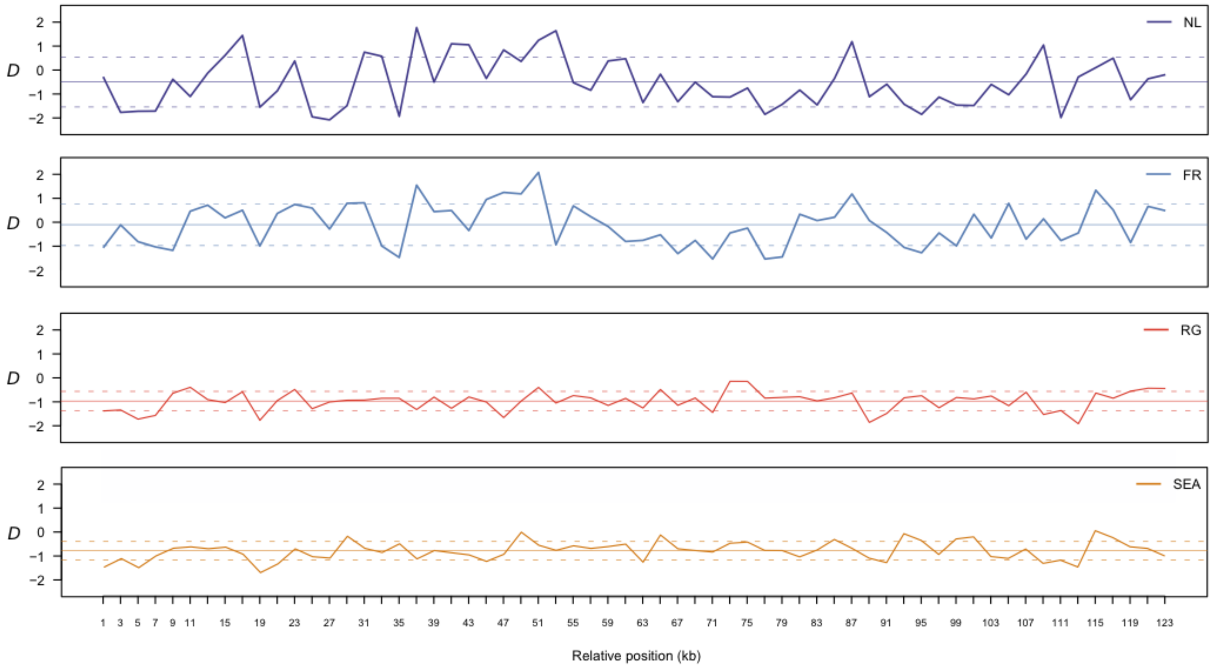

Figure S2 Tajima's *D* statistics. Tajima's *D* profiles along the 124 kb of interest are shown for the following populations: the Netherlands (NL), France (FR), Rwanda (RG), and Southeast Africa (SEA). The solid thin lines represent the corresponding mean value across the entire region, while dashed lines mark 1 SD above and below the corresponding mean.

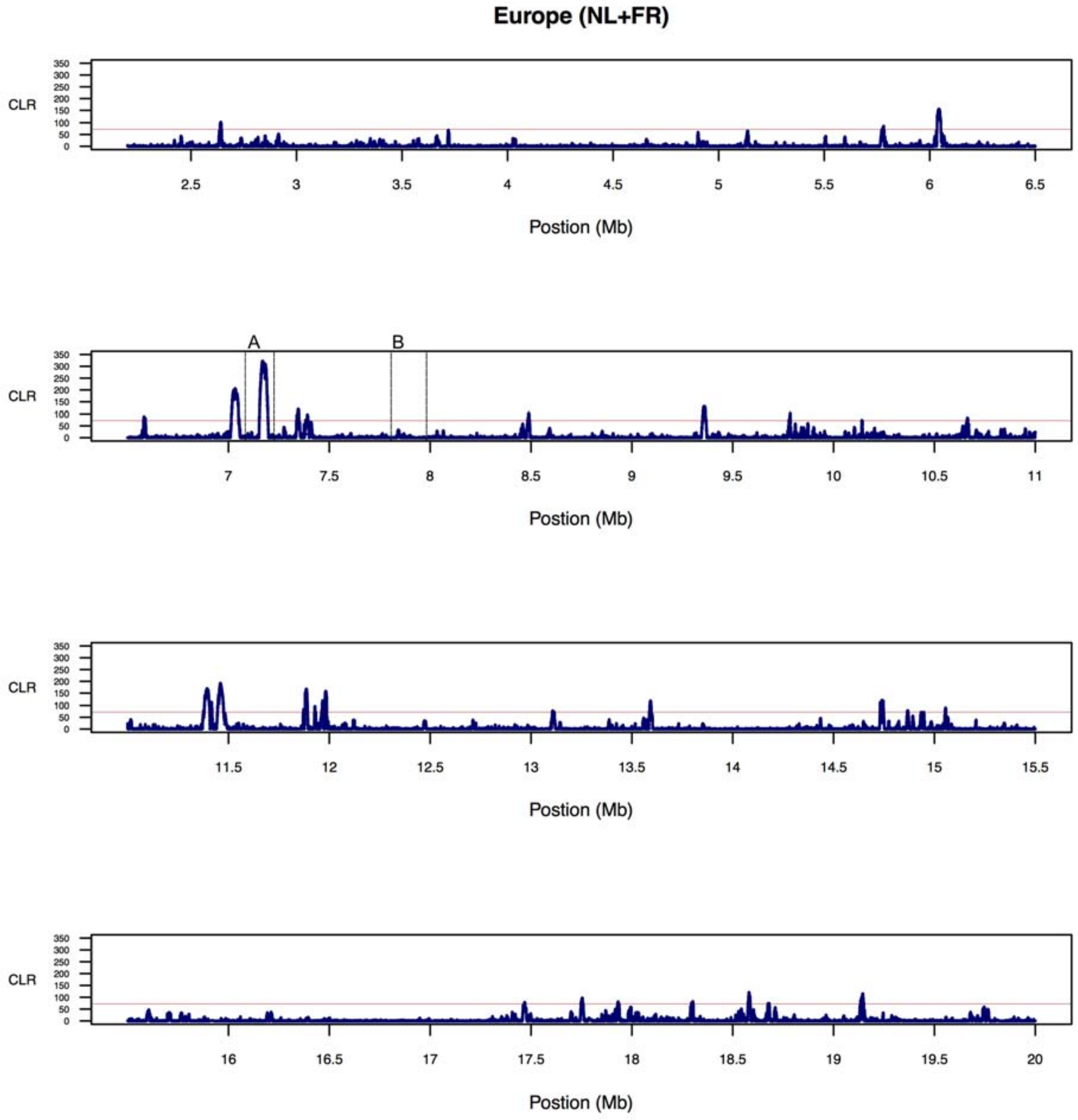

Figure S3 X-chromosome CLR profile for Europe. Composite likelihood ratio (CLR) test results for 18 Mb of a sample of 19 European (the Netherlands and French) *D. melanogaster* X-chromosomes. For this chromosome-wide test all categories (0 to *n*) of the SFS were included. The significance threshold at CLR=72 was obtained from simulated subgenomic datasets (see text and Figure S4). (A) indicates the interval with a CLR peak above 300 corresponding to that under deletion *Df(1)ED6906* (124 Kb long), also depicted in Figure 2A. (B) shows the Interval corresponding to that revealed by deletion *Df(1)C128* (131 Kb long). Note that this interval does not exhibit CLR peaks above the significance threshold.

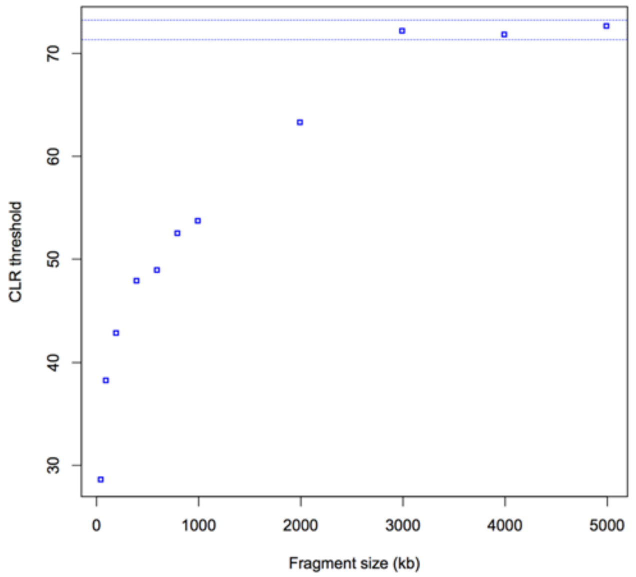

Figure S4 CLR thresholds *vs.* simulated fragment size. CLR thresholds, *i.e.* the top 5% CLR values of 100 simulated fragments of lengths from 5 to 5000 kb reach an asymptotic value around 72 at fragment size ≥3000 kb.

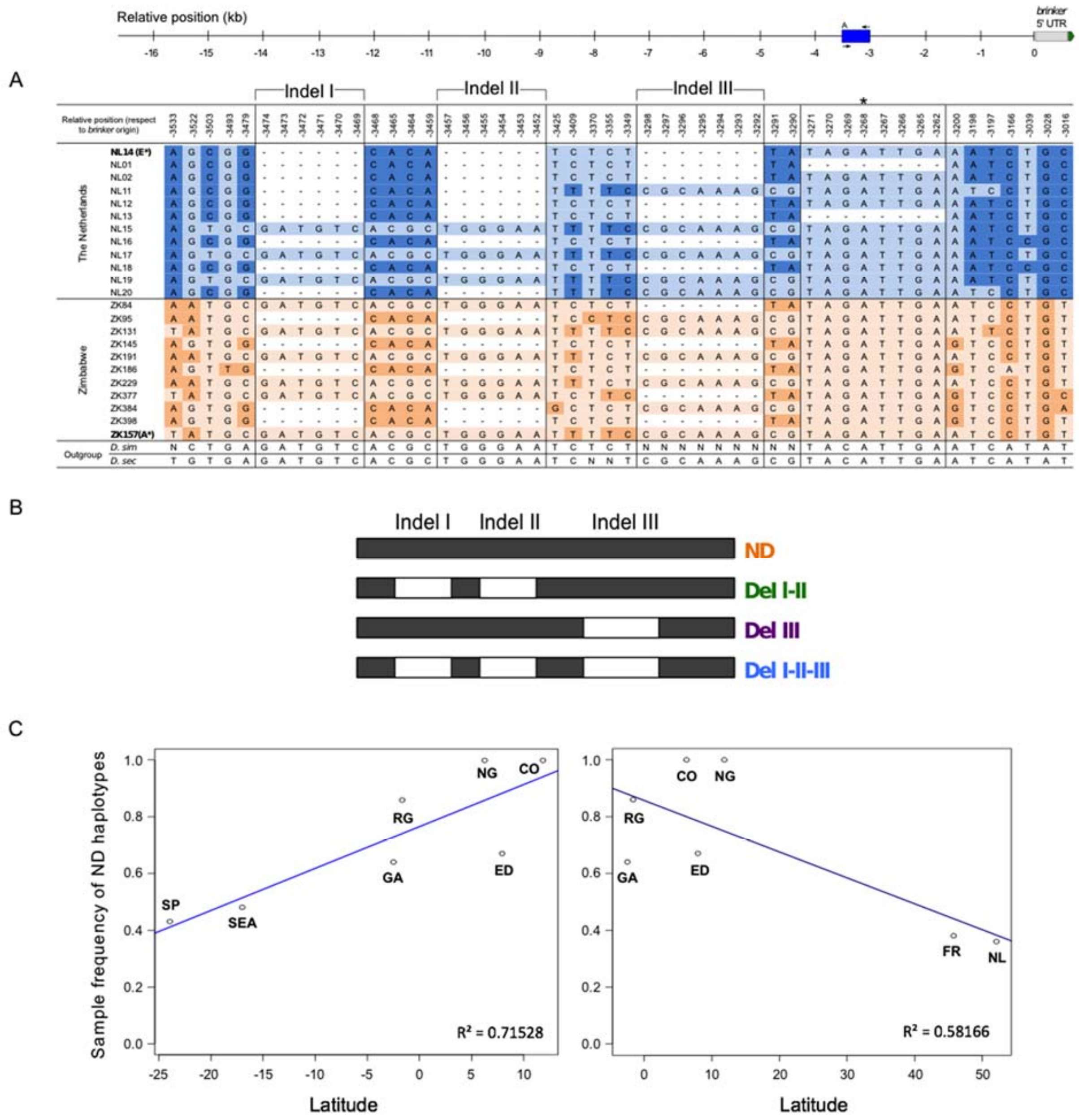

Figure S5 Putative *cis*-regulatory element upstream of *brinker.* A) Polymorphism table of a 534-bp fragment between relative positions -3,000 to -3,553 upstream of *brinker*. The figure depicts SNPs and structural variants (indels) of two *D. melanogaster* population samples from the Netherlands (NL) and Zimbabwe (ZK), including E* (top line) and A* (bottom line) plus two outgroups (*D. simulans* and *D. sechellia*). Light blue and orange indicate the inferred ancestral state of the SNP considering the two outgroups in NL and ZK, respectively, whereas darker tones of the same color represent the derived allele. Deletions are indicated in white background. Relative position -3,268 marked with an asterisk is highly associated with CCRT in the Raleigh population. B) Four haplotypes defined by the presence/absence of deletions and their numbers in the fragment. C) Frequency clines of the non-deletion haplotypes along a latitudinal gradient of *D. melanogaster* populations: the Netherlands (NL), France (FR), Nigeria (NG), Ethiopia (ED), Cameroon (CO), Gabon (GA), Rwanda (RG), Zambia + Zimbabwe + Malawi (SEA), and South Africa (SP).
