# Supplementary material for: Fine-Mapping and Selective Sweep Analysis of QTL for Cold Tolerance in Drosophila melanogaster
Source: G3 (Bethesda). 2014 Jun 26;4(9):1635–45. doi: 10.1534/g3.114.012757 (PMC4169155; doi:10.1534/g3.114.012757)
Supplement: Supporting Information [file supp_g3.114.012757_FigureS1.pdf]

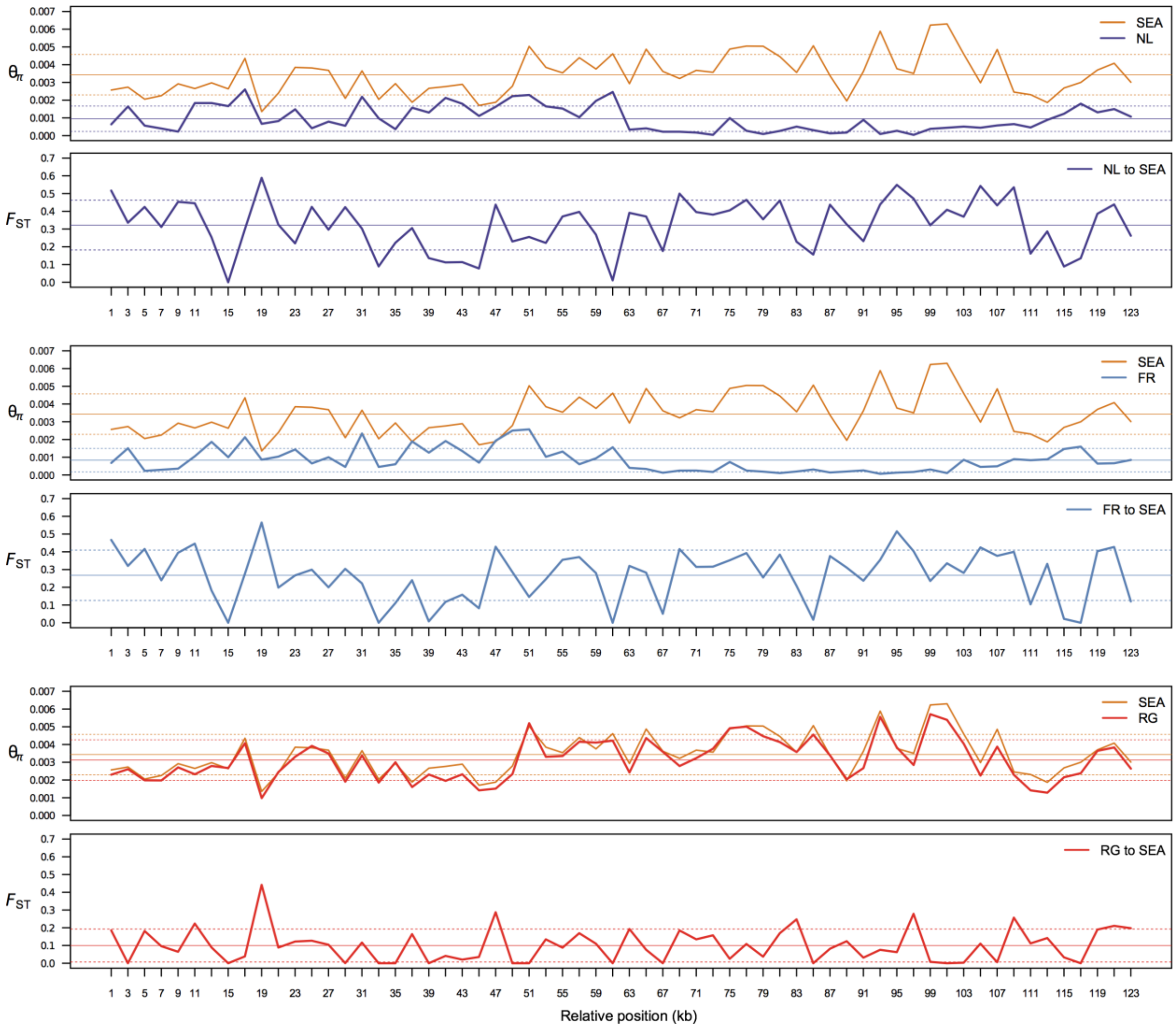

Figure S1 Polymorphism and between-population differentiation along the 124 kb of interest. Nucleotide diversity (θ_π) obtained for consecutive 2-kb long windows in four different populations: the Netherlands (NL), France (FR), Rwanda (RG) and a pool of Southeast African (SEA) lines sampled around Lake Kariba in Zimbawe and Zambia. This pool also includes lines from Malawi. The SEA profile is shown in all three θ_π panels for sake of comparison. Below each θ_π panel, inter-population differentiation profiles are plotted. Differentiation (F_{ST}) was calculated as normalized distance of Nei. Thin continous lines represent the average value for each summary statistic across the 62 windows, dashed lines represent 1 SD above and below the corresponding summary statistic mean.
