# Supplementary material for: Fine-Mapping and Selective Sweep Analysis of QTL for Cold Tolerance in Drosophila melanogaster
Source: G3 (Bethesda). 2014 Jun 26;4(9):1635–45. doi: 10.1534/g3.114.012757 (PMC4169155; doi:10.1534/g3.114.012757)
Supplement: Supporting Information [file supp_g3.114.012757_FigureS2.pdf]

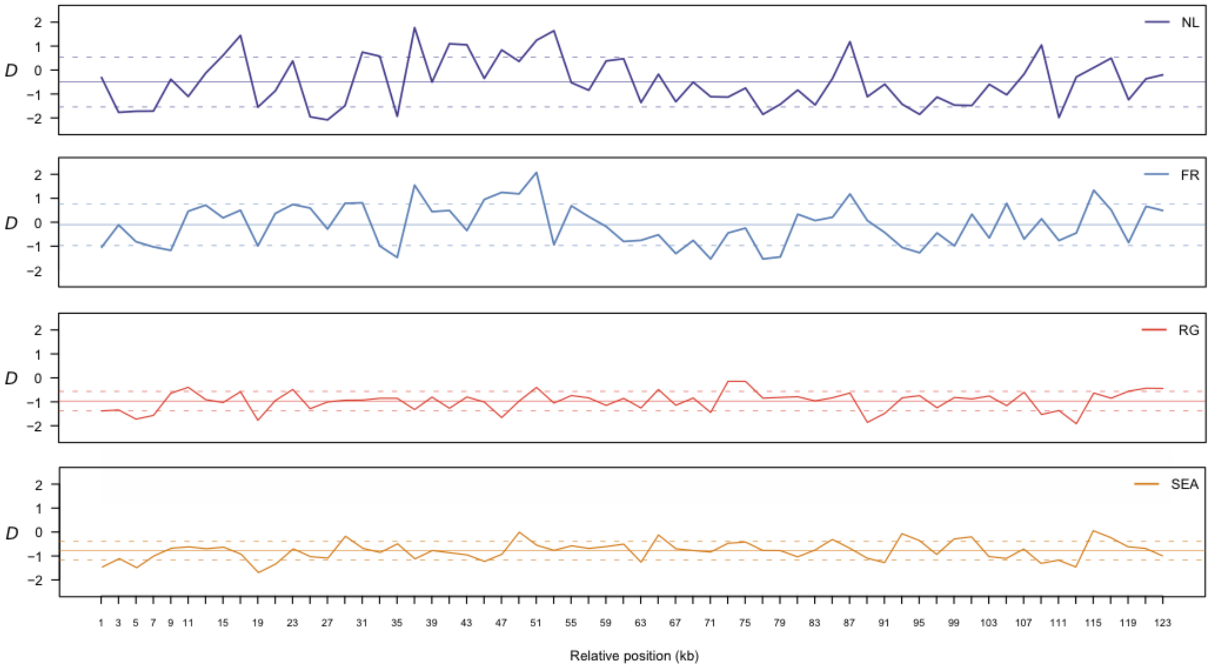

Figure S2 Tajima's *D* statistics. Tajima's *D* profiles along the 124 kb of interest are shown for the following populations: the Netherlands (NL), France (FR), Rwanda (RG), and Southeast Africa (SEA). The solid thin lines represent the corresponding mean value across the entire region, while dashed lines mark 1 SD above and below the corresponding mean.
