# Supplementary material for: Fine-Mapping and Selective Sweep Analysis of QTL for Cold Tolerance in Drosophila melanogaster
Source: G3 (Bethesda). 2014 Jun 26;4(9):1635–45. doi: 10.1534/g3.114.012757 (PMC4169155; doi:10.1534/g3.114.012757)
Supplement: Supporting Information [file supp_g3.114.012757_FigureS3.pdf]

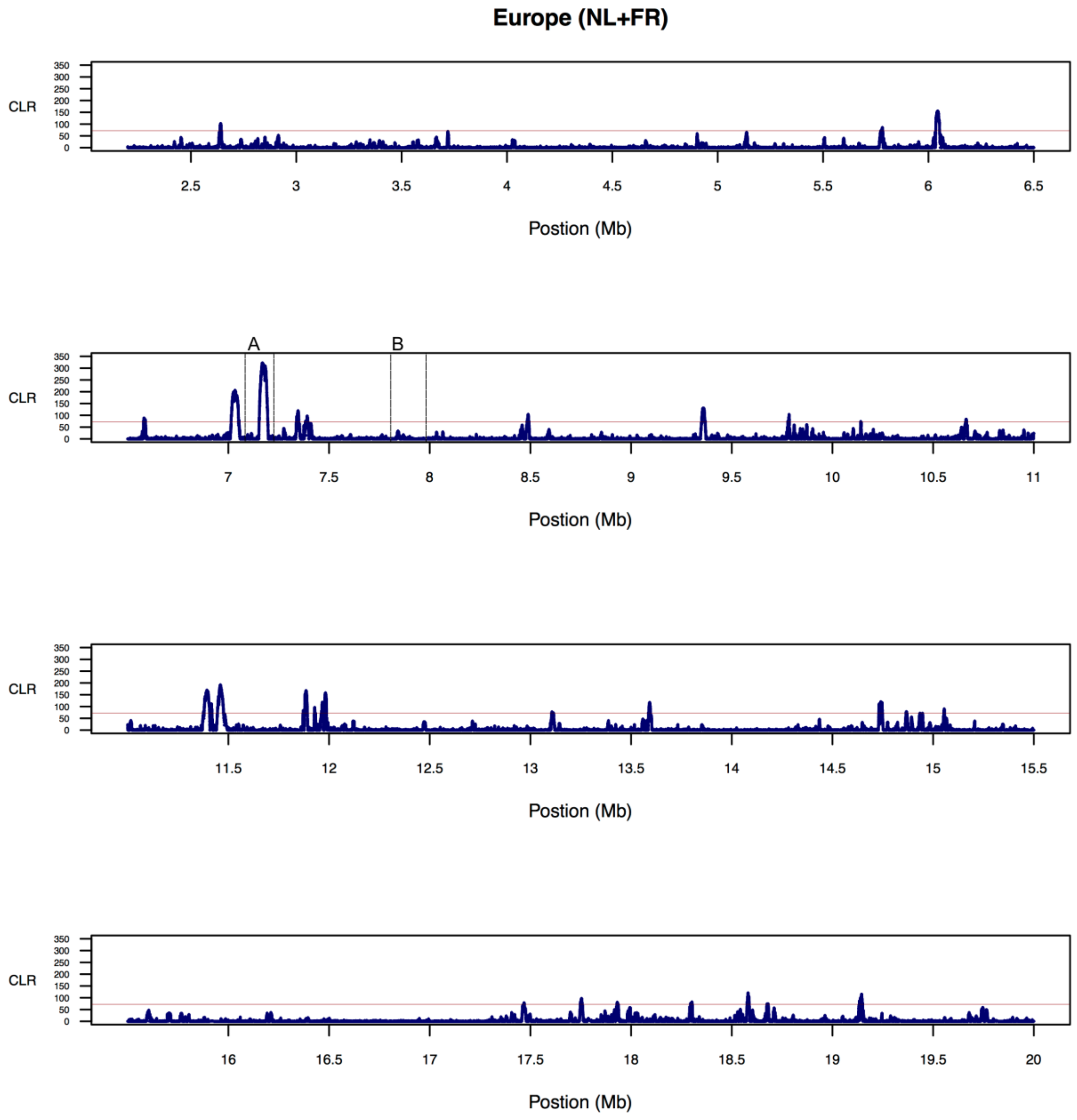

Figure S3 X-chromosome CLR profile for Europe. Composite likelihood ratio (CLR) test results for 18 Mb of a sample of 19 European (the Netherlands and French) *D. melanogaster* X-chromosomes. For this chromosome-wide test all categories (0 to *n*) of the SFS were included. The significance threshold at CLR=72 was obtained from simulated subgenomic datasets (see text and Figure S4). (A) indicates the interval with a CLR peak above 300 corresponding to that under deletion *Df(1)ED6906* (124 Kb long), also depicted in Figure 2A. (B) shows the Interval corresponding to that revealed by deletion *Df(1)C128* (131 Kb long). Note that this interval does not exhibit CLR peaks above the significance threshold.
