# Supplementary material for: Fine-Mapping and Selective Sweep Analysis of QTL for Cold Tolerance in Drosophila melanogaster
Source: G3 (Bethesda). 2014 Jun 26;4(9):1635–45. doi: 10.1534/g3.114.012757 (PMC4169155; doi:10.1534/g3.114.012757)
Supplement: Supporting Information [file supp_g3.114.012757_FigureS4.pdf]

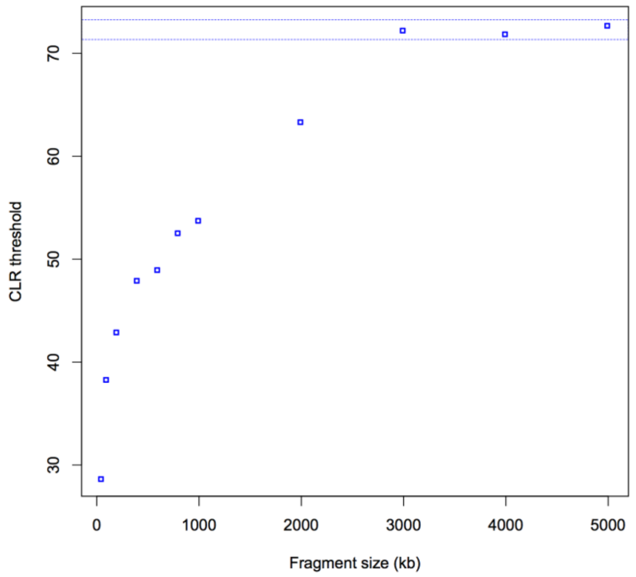

Figure S4 CLR thresholds *vs.* simulated fragment size. CLR thresholds, *i.e.* the top 5% CLR values of 100 simulated fragments of lengths from 5 to 5000 kb reach an asymptotic value around 72 at fragment size ≥3000 kb.
