# Supplementary material for: Fine-Mapping and Selective Sweep Analysis of QTL for Cold Tolerance in Drosophila melanogaster
Source: G3 (Bethesda). 2014 Jun 26;4(9):1635–45. doi: 10.1534/g3.114.012757 (PMC4169155; doi:10.1534/g3.114.012757)
Supplement: Supporting Information [file supp_g3.114.012757_FigureS5.pdf]

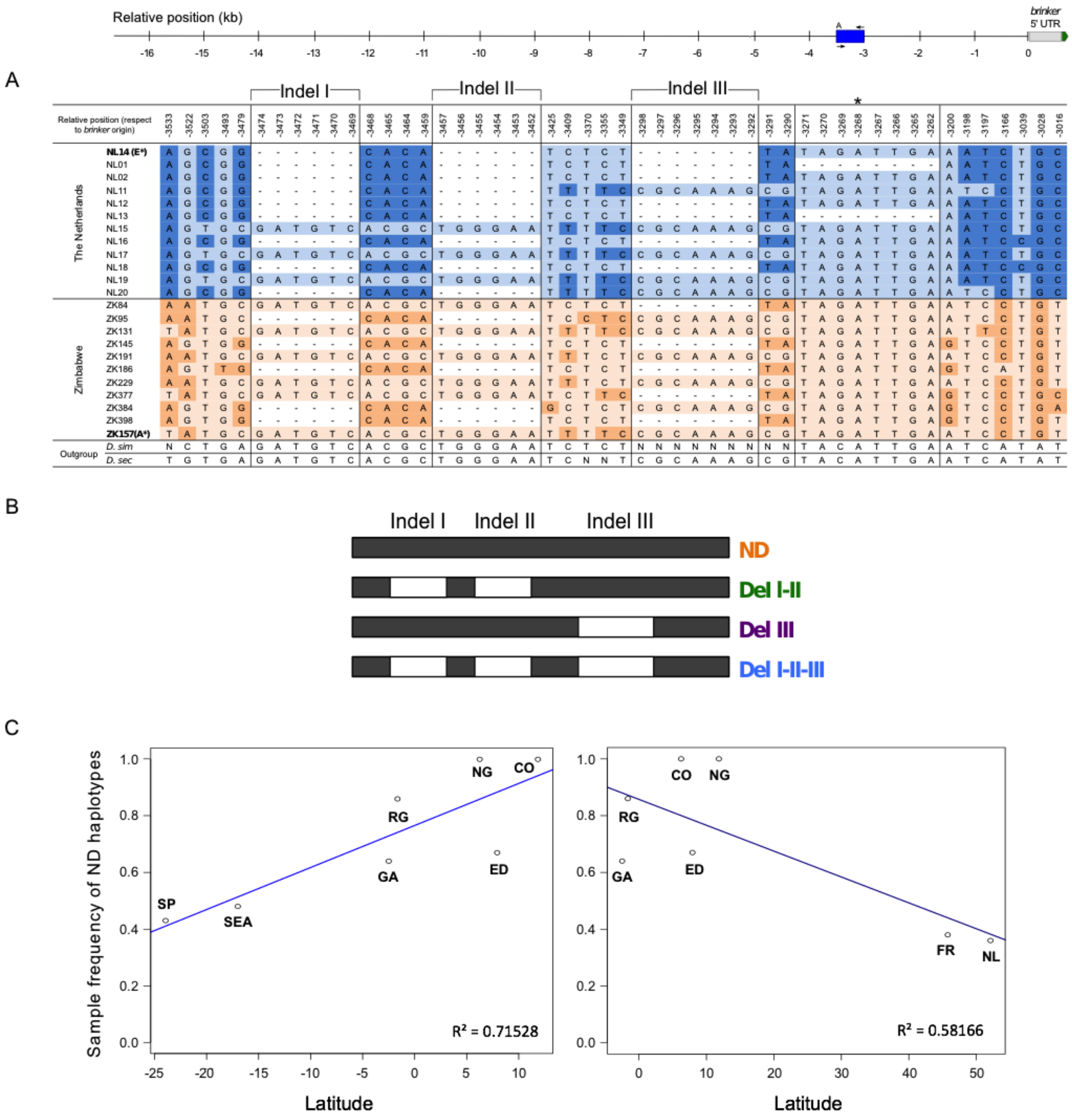

Figure S5 Putative *cis*-regulatory element upstream of *brinker.* A) Polymorphism table of a 534-bp fragment between relative positions -3,000 to -3,553 upstream of *brinker*. The figure depicts SNPs and structural variants (indels) of two *D. melanogaster* population samples from the Netherlands (NL) and Zimbabwe (ZK), including E* (top line) and A* (bottom line) plus two outgroups (*D. simulans* and *D. sechellia*). Light blue and orange indicate the inferred ancestral state of the SNP considering the two outgroups in NL and ZK, respectively, whereas darker tones of the same color represent the derived allele. Deletions are indicated in white background. Relative position -3,268 marked with an asterisk is highly associated with CCRT in the Raleigh population. B) Four haplotypes defined by the presence/absence of deletions and their numbers in the fragment. C) Frequency clines of the non-deletion haplotypes along a latitudinal gradient of *D. melanogaster* populations: the Netherlands (NL), France (FR), Nigeria (NG), Ethiopia (ED), Cameroon (CO), Gabon (GA), Rwanda (RG), Zambia + Zimbabwe + Malawi (SEA), and South Africa (SP).
